# Healthcare choices following mild traumatic brain injury in Australia

**DOI:** 10.1186/s12913-022-08244-3

**Published:** 2022-07-04

**Authors:** Jacinta Thorne, Shaun Markovic, HuiJun Chih, Elizabeth Thomas, Amanda Jefferson, Samar Aoun, Melinda Fitzgerald, Sarah Hellewell

**Affiliations:** 1 School of Allied Health, Faculty of Health Sciences, Curtin University, Bentley, WA 6102 Australia; 2 Perron Institute for Neurological and Translational Science, Nedlands, WA 6009 Australia; 3 Curtin Health Innovation Research Institute, Curtin University, Bentley, WA 6102 Australia; 4 Centre for Healthy Ageing, Health Futures Institute, Murdoch University, Murdoch, WA 6150 Australia; 5 Australian Alzheimer’s Research Foundation, Nedlands, WA 6009 Australia; 6 School of Population Health, Curtin University, Bentley, WA 6102 Australia; 7 Curtin Medical School, Faculty of Health Sciences, Curtin University, Bentley, WA 6102 Australia; 8 University of Western Australia, Crawley, WA 6009 Australia; 9 La Trobe University, Bundoora, VIC 3086 Australia

**Keywords:** Concussion, Mild traumatic brain injury, Epidemiology, Incidence, Population, Care choice

## Abstract

**Background:**

Accurate data on the types of healthcare people seek in the early stages following mild traumatic brain injury (mTBI) in Australia is lacking. We sought to investigate the types of healthcare people seek following mTBI, including seeking no care at all; ascertain the demographic, pre- and peri-injury factors, and symptom characteristics associated with the care that people access; and examine whether choice of care is associated with symptomatic recovery and quality of life.

**Methods:**

An online retrospective survey of Australians aged 18 to 65 years who had experienced a self-reported ‘concussion’ (mTBI) within the previous 18 months. Types of healthcare accessed were investigated, as well as those who did not seek any care. Data were analysed using frequency and percentages, chi-squared tests and logistic regression models.

**Results:**

A total of 201 respondents had experienced a self-reported ‘concussion’ but 21.4% of the respondents did not seek any care. Of the 183 respondents who sought healthcare, 52.5% attended a hospital Emergency Department, 41.0% attended a general practitioner and 6.6% accessed sports-based care. Compared to their counterparts, those who had a lower level of education (*p* = 0.001), had experienced previous mTBI (*p* = 0.045) or previous mental health issues (*p* = 0.009) were less likely to seek healthcare, whilst those who had experienced loss of consciousness (*p* = 0.014), anterograde (*p* = 0.044) or retrograde (*p* = 0.009) amnesia, and symptoms including drowsiness (*p* = 0.005), nausea (*p* = 0.040), and feeling slow (*p* = 0.031) were more likely to seek care. Those who did not seek care were more likely to recover within one month (AOR 4.90, 95%CI 1.51 – 15.89, *p* = 0.008), albeit the relatively large 95%CI warrants careful interpretation. Compared to seeking care, not seeking care was not found to be significantly associated with symptom resolution nor quality of life (*p* > 0.05).

**Conclusions:**

This study provides unique insight into factors associated with healthcare utilisation in the early stages following mTBI, as well as outcomes associated with choice of care, including not seeking care. Delivering targeted community education on the signs and symptoms of mTBI, and the advantages of seeking care following injury is an important step forward in the management of this challenging condition.

**Supplementary Information:**

The online version contains supplementary material available at 10.1186/s12913-022-08244-3.

## Introduction

Mild traumatic brain injury (mTBI), also known as concussion, has elicited increasing public concern and research attention over the past two decades [1]. mTBI can occur from a variety of mechanisms including falls, transport-related accidents, sporting incidents, assaults and military-related blast injuries, and can affect individuals of all ages [2]. Whilst the terminology suggests they are ‘mild’, the consequences can be significant, with an estimated 10–20% of individuals experiencing ongoing physical, cognitive and social issues for weeks, months or even years following a ‘mild’ injury [3]. There is also increasing concern that mTBI may have longer-term consequences for brain health, with potential associations observed with ongoing mental health issues [4–6] and neurodegenerative disorders such as dementia and Parkinson’s disease [7, 8]. For these reasons, mTBI is increasingly noted as a significant public health issue both in Australia and worldwide [1, 9].

To define the extent and scope of the mTBI issue, it is necessary to accurately determine its incidence and prevalence. Unfortunately, the true incidence of mTBI is challenging to determine, largely due to difficulty in quantifying head injury episodes within health systems, and in capturing those who do not present for medical attention [2, 10]. In 2004 the World Health Organization reported the incidence of hospital-treated mTBI at approximately 100–300 per 100,000 population but suggested that this was likely a vast under-estimation, and the true rate was likely to be more than 600 per 100,000 of the population, based upon USA household surveys [10]. The most recent data reported by the Australian Institute of Health and Welfare reported 22,710 hospitalisations for concussion in 2004/05 [11]. However, incidence rates calculated from hospital data alone are likely to considerably under-estimate the true numbers of people experiencing mTBI [12]. Australia has a unique healthcare structure in which there are both public and private hospitals, as well as privately owned and operated primary care general practices and allied health practices. There is currently no centralised health information system or insurance scheme linking these healthcare settings [13], and thus it is not currently possible to obtain comprehensive incidence data for individuals who seek care for a mTBI outside of the hospital setting in Australia [14].

More difficult to ascertain is the number of individuals who sustain a mTBI but are not medically diagnosed, referred to as the “silent minority” [15]. There may be several reasons why individuals do not present for care. Firstly, many individuals are not aware that they have sustained a mTBI [16] or their symptoms are subtle and they chose to “watch and wait” rather than seeking care immediately [12, 17]. Secondly, individuals may be unwilling or unable to report their injuries – for example, because of socio-cultural norms within a sporting context where athletes may not want to report their injuries to coaching staff or peers [18, 19]. Similarly, in situations of intimate partner violence a victim may be unable to access healthcare resources or may be reluctant to report the incident [20, 21]. Finally, many mTBI’s remain undiagnosed because of considerable variability in recognition and diagnosis by medical practitioners, or as a result of other co-existing or more severe injuries taking precedence [17].

This gap in the knowledge of the estimated number of people who seek no care at all may be filled by utilising population-based surveys [10]. Gordon (2019) used data gathered as part of the Canadian Community Health Survey and determined that of 1749 people surveyed who had experienced a mTBI within the previous 12 months, 21.9% had not received medical attention, whilst 69% had attended a hospital emergency department and 11% sought care at a doctor’s office [15]. A telephone-based survey of people in Colorado, USA found that of those who reported a mTBI, approximately 30% did not seek any medical care [22], and 42% of respondents from an USA internet-based survey did not seek care following TBI [23]. To our knowledge, there is currently no available data on the types of healthcare people access following mTBI in Australia, particularly with regards to those who do not seek care at all. Improving our knowledge of healthcare seeking behaviours following mTBI will enhance our ability to account for all potential cases, and this information may inform future studies which seek to determine the incidence of mTBI in Australia.

Thus, the objectives of our study were to investigate the types of healthcare people seek in the early stages following a mTBI, including seeking no care at all; and to ascertain demographic factors, pre- and peri-injury factors, and symptom characteristics associated with the healthcare that people access. In particular, we were interested in those respondents who did not seek care, as it is generally difficulty to capture data on this cohort using traditional research methods. Consequently, the final objective of this study was to explore the association between healthcare (seeking care or not seeking care) and recovery, including symptom resolution and quality of life.

## Methods

### Study design

This study utilised data collected as part of a larger survey entitled *“Recovery Experiences Following Concussion”*, a retrospective online survey of Australians, aged 18 to 65 years, who reported experiencing a mTBI within the past 18 months from any cause, including falls, transport accidents, sporting injuries or assaults. The *“Recovery Experiences Following Concussion”* survey incorporated a comprehensive array of topics related to mTBI recovery in an Australian population, including the types of healthcare that patients access following mTBI, psychosocial and lifestyle factors, exercise following mTBI, the influence of personality, resilience and coping styles, and the longer term effects of mTBI on quality of life, wellbeing and productivity. In order to address the objectives of the current study, data were drawn from the following sections of the survey (see Supplementary Table 1 for full survey content):

- 1. Respondent demographic information, such as age, sex, state of residence, education level, and income.
- 2. Choice of initial healthcare following mTBI, which included the option to select that no medical care was accessed and the reasons for this.
- 3. Injury characteristics and circumstances; including injury mechanism and description, loss of consciousness, retrograde and anterograde amnesia (memory loss for events immediately before and after the mTBI occurred respectively), symptoms experienced, symptom resolution and time to symptom resolution.
- 4. Relevant medical history, including previous concussions and previous mental health issues.
- 5. Effect of mTBI on quality of life.

The survey was designed through consultation amongst research group members to ensure good content validity, and consumer input was obtained to verify readability and comprehension of the survey questions. Ethics approval was obtained from the Curtin University Human Research Ethics Committee (Approval Number HRE2020-0536). Potential participants were provided with information about the research prior to commencing the survey and all eligible respondents gave their digital informed consent to participate. All surveys were completed in anonymised format.

### Sample recruitment

Respondents were recruited over a three-month period commencing in November 2020. The survey was hosted and distributed using Qualtrics (Qualtrics, Provo, Utah, 2021) an independent panel aggregator with extensive experience in academic survey design. The survey was distributed via email invitation to Qualtrics’ network of third-party panel providers based in Australia, who have pre-existing databases of potential survey respondents interested in completing surveys for corporate and academic research. Respondents received a non-monetary incentive from Qualtrics or their panel providers on completion of the survey. To minimise the likelihood of self-selection bias, survey invitations did not include specific details about the survey content. Respondents were asked to complete a series of three pre-screening eligibility questions in which the topic of the survey was deliberately masked, and which asked, “Please select which of the following injury scenarios you may have experienced in the past 18 months?” Respondents could select responses including lower back injury, muscle or joint sprain/strain, concussion, fracture or broken bone, or no injury. Participants were only included if they were an Australian citizen or permanent resident, were aged between 18 and 65 years, and identified as having sustained a concussion injury in the past 18 months. Concussion status was based upon the respondent’s self-report and was defined in a further screening question as:

“An incident likely to lead to a head injury – including a knock to your head, or an impact to your body which resulted in force being transferred to your head, and then experienced signs or symptoms related to that incident. You may have also experienced one or more of the following:

1. Any loss of consciousness (Were you “knocked out” or did you “black out”?)*
2. Altered mental state at the time of the accident (Were you dazed, disoriented or confused? Did you “see stars” at the time of injury?)
3. Experience any memory loss for events immediately before or after the accident (Do you have any memory loss around the time of injury – before or after?)
4. Any symptoms such as headache, dizziness, fogginess (or other symptoms) around the time of the incident?

“*You do not need to have lost consciousness or been 'knocked out' to have had a concussion.”

### Data quality assessment

Following collection, the data were first screened for quality by Qualtrics, and subsequently were independently screened by three members of the study research group (SH, JT, SM) and a consensus was reached on those responses that were deemed poor quality. Survey responses were removed based upon the following criteria as recommended by Qualtrics: incomplete survey responses, duplicate responses, ambiguous or nonsensical text, incongruent responses (e.g. reporting multiple different injury mechanisms), completing the survey abnormally fast (less than 8 min), or responses selected in a straight-line pattern for more than 20% of the survey.

### Study measures

The primary outcome measure of interest for this study was the type of healthcare accessed in the early stages following mTBI. Survey respondents were asked, “When you first experienced your most recent concussion, where did you seek medical care?” Respondents were able to select more than one healthcare option and thus could be included in more than one category. The responses were then re-grouped into the following four main categories: 1. No Care; 2. Hospital Emergency Department – which combined the responses for public and private hospital emergency departments; 3. Primary care practitioners – included those who selected “General practitioner (GP)” and “Urgent Care or After-Hours Care”; and 4. Sports-based care – included those who selected “Sports Doctor/Physician”, and “Sports Club Personnel (Doctor, Physio, and Sports Trainer)”. When “Other” was selected as a type of care response, the open field text response provided was used to re-categorise the response into the most appropriate care category. Respondents who indicated they did not seek care were subsequently prompted to provide reasons for this decision, including an open field option. Location of initial healthcare was then stratified according to pre- and peri-injury factors such as previous mTBI, relevant aspects of past medical history, mechanism of injury, change in conscious state, memory loss and symptom presentation.

Symptom presentation was assessed using a modified version of the 22-item Post-Concussion Symptom Scale (PCSS) [24]. The PCSS is normally structured using a six-point Likert scale to provide both the number of symptoms experienced out of a total of 22, and a total symptom severity score out of 132. As this was a retrospective survey respondents were asked to select the symptoms they had experienced in the early stages following mTBI, but were not required to rate their symptom severity as we felt that recall for this detail may not be accurate. Thus early symptom presentation in this study is presented as the total number of symptoms endorsed out of a total of 22, and has also been re-categorised into lower (1 to 5) or higher (6 or more) number of symptoms, based upon the average number of symptoms of the cohort. Quality of life was evaluated using the six item short-form Quality of Life after Brain Injury Scale – Overall Scale (QOLIBRI-OS), which has good test–retest reliability (0.81) and validity (*r* = 0.87) [25]. Responses were converted to a percentage score, and then re-categorised as either a higher quality of life (≥ 75%) or lower quality of life (< 75%) QOLIBRI-OS score, based upon median reference values from the general population [26]. Time to symptom resolution was re-categorised as a binary variable of less than or greater than one month, as one month is widely recognised as a ‘normal’ timeframe for recovery following mTBI [3, 27]. Other variables utilised in this analysis are included in Supplementary Table 1, and have been re-categorised as detailed for ease of interpretation where appropriate.

### Statistical analysis

All data obtained from this study were analysed using IBM SPSS Statistics Data Editor Version 27. Descriptive statistics were calculated for demographic, injury-related characteristics, symptom resolution and quality of life for all respondents. Mean and standard deviations were reported for continuous variables (all of the continuous variables displayed normal distribution), whilst frequency and percentages were reported for categorical variables. Column percentages were provided for each dependent variable and add to 100%. Associations between factors and care seeking were assessed using chi-squared tests, or Fisher’s exact test when expected cell counts were less than 5. When open-text responses were provided the responses were coded to common categories and then reported as a count and percentage. As our initial analysis revealed a number of associations of interest for the group of respondents who did not seek care, we were then particularly interested in whether presenting symptoms may influence recovery in those who did not seek care. Logistic regression models were used to further examine the relationship between choice of healthcare (No Care/Care) and Symptom Resolution (yes/no), Time to Symptom Resolution (< 1 month/1 month or longer) and quality of life (QOLIBRI-OS score higher/lower). Logistic regression results are presented as crude and adjusted odds ratios (aOR) with 95% confidence intervals, where Symptom Resolution was adjusted for type of initial symptoms (headache, nausea, dizziness, drowsiness, feeling slow, feeling foggy); Time to Symptom Resolution was adjusted for number of initial symptoms (PCSS total score) and type of initial symptoms; and the QOLIBRI-OS score was adjusted for number of initial symptoms (PCSS total score), type of symptoms, and years of education. We adjusted for potential confounders in the models to minimise confounding bias. The potential confounders were determined according to the criteria outlined by Rothman et al. (2008) [28] as (i) related to the independent variable, (ii) related to the outcome variable, but (iii) not an intermediate of the independent and outcome variable. Since the outcome variables of the models were different, the confounders were different for the models. Significance was set at an alpha level of 0.05.

## Results

In total, 4505 people were invited to participate in the survey via email link. Of these, 274 respondents completed the survey and 73 survey responses were removed following data quality assessment outlined in the Methods. In alignment with the objectives of this study, the resultant data are presented in two parts. In the first part the outcome variable of interest is the type of healthcare accessed by each respondent and the association of various demographic, injury-related factors and symptom presentation with choice of care. We were particularly interested in those respondents who did not seek care, and thus have included more detailed analysis comparing No Care to Care. The second part of the results considers the relationship between healthcare choice and recovery, including whether symptoms had resolved, the time taken for symptom resolution, and the impact of mTBI on the respondent’s quality of life.

### Demographic characteristics

Of the 201 survey responses analysed, 53.2% of respondents were female and 46.3% were male (Table 1). The mean age of respondents was 37.7 years. Most of the cohort (78.6%) sought some form of healthcare in the early stages after mTBI, however, of particular interest, 21.4% did not seek any care. Characteristics including age, sex, state of residence, employment status and household income were comparable between those who did or did not present for healthcare (*p* > 0.05). However there was a significant association between a respondent’s level of education and whether or not they sought care (*p* = 0.001). Of those who *did not* seek care, 25.6% had the lowest level of education (had completed secondary education to Year 10), whereas only 2.5% who *did* seek care had the lowest level of education. In other words, of those who had the lowest level of education 11 out of 15 (73.3%) did not seek care. In comparison, only 5 out of 34 (14.7%) respondents in the highest level of education group (post-graduate level) did not seek care.

**Table 1:** Demographic characteristics and association between those who did or did not seek initial healthcare

	**Whole Cohort (*n* = 201)**	**No Care (*n* = 43)**	**Care (*n* = 158)**	***P*-value**^a^
**Age** *mean (SD)*	37.7 (12.3)	40.1 (11.1)	37.0 (12.6)	0.150
**Age Categories** *n (%)*				0.100
18–25	29 (14.4)	3 (7.0)	26 (16.5)	
26–35	79 (39.3)	15 (34.9)	64 (40.5)	
36–45	37 (18.4)	19 (23.3)	27 (17.1)	
46–55	34 (16.9)	12 (27.9)	22 (13.9)	
56–65	22 (10.9)	3 (7.0)	19 (12.0)	
**Sex** *n (%)*				0.392^b^
Female	107 (53.2)	27 (62.8)	80 (50.6)	
Male	93 (46.3)	16 (37.2)	77 (48.7)	
Other	1 (0.5)	0 (0.0)	1 (0.6)	
**State of Residence** *n (%)*				0.737^b^
ACT	9 (4.5)	0 (0.0)	9 (4.5)	
NSW	54 (26.9)	12 (27.9)	42 (26.6)	
NT	1 (0.5)	0 (0.0)	1 (0.6)	
QLD	39 (19.4)	9 (20.9)	30 (19.0)	
SA	19 (9.5)	3 (7.0)	16 (10.1)	
TAS	7 (3.5)	2 (4.7)	5 (3.2)	
VIC	53 (26.4)	14 (32.6)	39 (24.7)	
WA	19 (9.5)	3 (7.0)	16 (10.1)	
**Years of Education** *mean (SD)*	14.3 (2.2)	13.5 (2.6)	14.5 (2.1)	**0.016**
**Educational Status** *n (%)*				**0.001**
Secondary education to Year 10	15 (7.5)	11 (25.6)	4 (2.5)	
Secondary education to Year 12	29 (14.4)	3 (7.0)	26 (16.5)	
Vocational education/training	29 (14.4)	6 (14.0)	23 (14.6)	
Diploma/Advanced Diploma	19 (9.5)	4 (9.3)	15 (9.5)	
Bachelor degree	75 (37.3)	14 (32.6)	61 (38.6)	
Post-graduate studies	34 (16.9)	5 (11.6)	29 (18.4)	
**Employment** *n (%)*				0.154
Full-time	104 (51.7)	18 (41.9)	86 (54.4)	
Self-employed	16 (8.0)	5 (11.6)	11 (7.0)	
Part-time	42 (20.9)	7 (16.3)	35 (22.2)	
Home Duties	9 (4.5)	4 (9.3)	5 (3.2)	
Currently not working	30 (14.9)	9 (20.9)	21 (13.3)	
**Household Income** *n (%)*				0.360
$1—$49,999	42 (20.9)	12 (27.9)	30 (19.0)	
$50,000—$99,999	77 (38.3)	19 (44.2)	58 (36.7)	
$100,000—$149,999	46 (22.9)	7 (16.3)	39 (24.7)	
$150,000—$199,999	24 (11.9)	4 (9.3)	20 (12.7)	
$200,000 or more	12 (6.0)	1 (2.3)	11 (7.0)	
**Household Income** *n (%)*				0.052
Less than $100,000	119 (59.2)	31 (72.1)	88 (55.7)	
$100,000 or greater	82 (40.8)	12 (27.9)	70 (44.3)	

^a^Difference in age and years of education between care groups was assessed using independent samples t-test; associations between categorical demographic variables and care groups were assessed using Chi-squared tests or Fisher’s Exact tests^b^ when expected cell counts were less than 5

### Relationship between injury-related (pre- and peri-injury) factors and choice of healthcare

Table 2 reports frequencies and percentages for each category of care type. As respondents could be included in more than one category the total frequency exceeded the number of respondents (201). Of those respondents who had sought healthcare, the largest percentage of respondents had attended a hospital emergency department (52.5%), followed by primary care practitioners (41.0%) and sports-based care (6.6%).

**Table 2:** Frequency and percentage of pre-and peri-injury factors by choice of healthcare^a^

	**Whole Cohort** (*n* = 201)	**No Care** (*n* = 43)	**Hospital ED** (*n* = 96)	**Primary Care** (*n* = 75)	**Sports-based Care** (*n* = 12)
**Healthcare Visits**^b^ *n (%)*	183	-	96 (52.5)	75 (41.0)	12 (6.6)
**Pre-Injury Factors**					
**Age** *Mean (SD)*	37.7 (12.3)	40.1 (11.1)	38.3 (12.9)	35.5 (12.4)	29.7 (4.6)
**Sex** *n (%)*					
Female	107 (53.2)	27 (62.8)	48 (50.0)	40 (53.3)	2 (16.7)
Male	93 (46.3)	16 (37.2)	47 (49.0)	34 (45.3)	10 (83.3)
Other	1 (0.5)	0 (0.0)	1 (1.0)	1 (1.3)	0 (0.0)
**Previous mTBI** *n (%)*					
Yes	60 (29.9)	16 (37.2)	28 (29.2)	23 (30.7)	4 (33.3)
No	133 (66.2)	23 (53.5)	66 (68.8)	50 (66.7)	8 (66.7)
Unsure	8 (4.0)	4 (9.3)	2 (2.1)	2 (2.7)	0 (0.0)
**Previous Mental Health Issues** *n (%)*					
Yes	69 (34.3)	22 (51.2)	24 (25.0)	31 (41.3)	2 (16.7)
No	132 (65.7)	21 (48.8)	72 (75.0)	44 (58.7)	10 (83.3)
**Previous Learning Disorders** *n (%)*					
Yes	18 (9.0)	4 (9.3)	11 (11.5)	7 (9.3)	2 (16.7)
No	183 (91.0)	39 (90.7)	85 (88.5)	68 (90.7)	10 (83.3)
**Previous Migraine** *n (%)*					
Yes	44 (21.9)	10 (23.3)	20 (20.8)	18 (24.0)	2 (16.7)
No	157 (78.1)	33 (76.7)	76 (79.2)	57 (76.0)	10 (83.3)
**Previous Sleep Disorders** *n (%)*					
Yes	37 (18.4)	5 (11.6)	19 (19.8)	13 (17.3)	3 (25.0)
No	164 (81.6)	38 (88.4)	77 (80.2)	62 (82.7)	9 (75.0)
**Peri-Injury Factors**					
**Mechanism of Injury** *n (%)*					
Sport-related	78 (38.8)	14 (32.6)	37 (38.5)	28 (37.3)	10 (83.3)
Non-sport Related	123 (61.2)	29 (67.4)	59 (61.5)	47 (62.7)	2 (16.7)
**Non-Sport Type** *n (%)*					
Fall	84 (68.3)	17 (58.6)	37 (62.7)	35 (74.5)	1 (50.0)
Transport Accident	14 (11.4)	1 (3.4)	11 (18.6)	5 (10.6)	1 (50.0)
Assault	13 (10.6)	5 (17.2)	6 (10.2)	5 (10.6)	0 (0.0)
Other	12 (9.8)	6 (20.7)	5 (8.5)	2 (4.3)	0 (0.0)
**Loss of Consciousness** *n (%)*					
Yes	112 (55.7)	16 (37.2)	63 (65.6)	42 (56.0)	8 (66.7)
No	71 (35.3)	23(53.5)	24 (25.0)	25 (33.3)	3 (25.0)
Unsure	18 (8.9)	4 (9.3)	9 (9.4)	8 (10.7)	1 (8.3)
**Anterograde Amnesia** *n (%)*					
Yes	78 (38.8)	11 (25.6)	46 (47.9)	32 (42.7)	5 (41.7)
No	97 (48.2)	28 (65.1)	40 (41.7)	30 (40.0)	5 (41.7)
Unsure	26 (12.9)	4 (9.3)	10 (10.4)	13 (17.3)	2 (16.7)
**Retrograde Amnesia** *n (%)*					
Yes	51 (25.4)	6 (14.0)	28 (29.2)	26 (34.7)	2 (16.7)
No	13 (66.2)	29 (67.4)	63 (65.6)	44 (58.7)	10 (83.3)
Unsure	17 (8.4)	8 (18.6)	5 (5.2)	5 (6.7)	0 (0.0)
**PCSS Total Number of Symptoms** *Mean (SD)*	5.7 (3.6)	4.9 (3.3)	6.0 (3.4)	6.6 (4.4)	6.2 (4.4)
**PCSS Number of Symptoms** *n (%)*					
1 to 5	106 (60.9)	29 (72.5)	43 (53.1)	35 (56.6)	6 (66.7)
6 or more	68 (39.1)	11 (27.5)	38 (46.9)	27 (43.5)	3 (33.3)

^a^Respondents were able to select more than one choice of initial care, and thus frequencies may add to greater than the total sample size of 201. Column percentages are provided for each category of care choice and add to 100%

^b^‘Healthcare visits’ was calculated based upon the total number of presentations for initial medical care (i.e. hospital ED’s, primary health care and sports-based care)

Differences from the expected frequencies were noted predominantly for those who did not seek care, and thus these have been explored in further detail in Table 3. Of the pre-injury factors considered, those who had experienced a previous mTBI (*p* = 0.045) and those who had experienced previous mental health issues (*p* = 0.009) were significantly more likely *not* to seek healthcare (Table 3). No relationship was identified between previous learning disorders, migraine or sleep disorders and the type of care accessed (*p* > 0.05). Of the peri-injury factors investigated, more who attended sports-based care were male (83% male compared to 17% female) (Table 2). No association was noted between sport-related injuries compared to non-sport related injuries and the type of care they accessed (*p* > 0.05) (Table 3). However, when the type of non-sport related injury was further clarified, 71%, 14% and 9% of those who sought care had a fall, experienced a transport accident or assault, respectively; whilst 59%, 3% and 17% of those who did not seek care had a fall, experienced a transport accident or assault (*p* = 0.031). Of note, six respondents reported mTBI as a result of assault during an episode of intimate partner violence, and three of these respondents did not seek any medical care. Reasons given for not seeking care included “Was not allowed by partner” (1 of the 3 respondents) and “I didn’t want others to know I had experienced a concussion” (2 of the 3 respondents).

**Table 3:** Association between pre-injury and peri-injury characteristics and choice of care (No Care/Care)

	**Whole Cohort** (*n* = 201)	**No Care** (*n* = 43)	**Care** (*n* = 158)	***P*-value**^a^
**Total** *n (%)*	201	43 (21.4)	158 (78.6)	
**Pre-Injury Factors**				
**Previous mTBI**				
Yes	60 (29.9)	16 (37.2)	44 (27.8)	**0.045**
No	133 (66.2)	23 (53.5)	110 (69.6)	
Unsure	8 (4.0)	4 (9.3)	4 (2.5)	
**History of Mental Health Issues**				
Yes	69 (34.3)	22 (51.2)	47 (29.7)	**0.009**
No	132 (65.7)	21 (48.8)	111 (70.3)	
**Peri-Injury Factors**				
**Mechanism of Injury** *n (%)*				
Sport-related	78 (38.8)	14 (32.6)	64 (40.5)	0.343
Non-sport Related	123 (61.2)	29 (67.4)	94 (59.5)	
**Non-Sport Type**				
Fall	84 (68.3)	17 (58.6)	67 (71.3)	**0.031**^b^
Transport Accident	14 (11.4)	1 (3.3)	13 (13.8)	
Assault	13 (10.6)	5 (17.2)	8 (8.5)	
Other	12 (9.8)	6 (20.7)	6 (6.4)	
**Loss of Consciousness** *n (%)*				
Yes	112 (55.7)	16 (37.2)	96 (60.8)	**0.014**
No	71 (35.3)	23(53.5)	48 (30.4)	
Unsure	18 (8.9)	4 (9.3)	14 (8.9)	
**Anterograde Amnesia** *n (%)*				
Yes	78 (38.8)	11 (25.6)	67 (42.2)	**0.044**
No	97 (48.2)	28 (65.1)	69 (43.7)	
Unsure	26 (12.9)	4 (9.3)	22 (13.9)	
**Retrograde Amnesia** *n (%)*				
Yes	51 (25.4)	6 (14.0)	45 (28.5)	**0.009**
No	133 (66.2)	29 (67.4)	104 (65.8)	
Unsure	17 (8.4)	8 (18.6)	9 (5.7)	

^a^Associations between pre-injury and peri-injury variables and care groups were assessed using Chi-squared tests, or Fisher’s Exact tests^b^ when expected cell counts were less than 5. Column percentages are provided for each category of care choice and add to 100%

In this survey sample, 55.7% had experienced a loss of consciousness (LOC) and 56.3% of those who suffered LOC attended a hospital ED, followed by primary care (37.5%) and sports-based care (7.1%) (Table 2). Surprisingly, 14.3% of people who had experienced a LOC did not seek any healthcare. Respondents were significantly more likely to seek some form of care if they had experienced LOC (*p* = 0.014) (Table 3). Similarly, respondents were significantly more likely to seek some form of care if they experienced either anterograde or retrograde amnesia (*p* = 0.044 and *p* = 0.009 respectively) (Table 3).

### Relationship between symptom presentation and choice of initial healthcare

Only those respondents whose symptoms had resolved were asked about their initial symptoms using the PCSS, and thus data from 174 respondents are included in this analysis. The mean number of symptoms reported on the PCSS was 5.7 ± 3.6 (Table 4). The mean PCSS score for those who did not seek care (4.9 ± 3.3) was slightly lower than those who did seek care (5.9 ± 3.6), although this did not reach statistical significance (*p* = 0.054) (Table 4). Of those respondents who did not seek care, 72.5% reported five or less symptoms in the early time period following their mTBI. While not statistically significant, it would seem that those who experienced a lower symptom burden were less likely to seek care. Each symptom was also considered separately, and whilst the PCSS includes a list of 22 symptoms, only the top five most frequently reported symptoms have been included in this analysis. Those who experienced drowsiness (*p* = 0.005), nausea (*p* = 0.040), and feeling ‘slow’ (*p* = 0.031) were significantly more likely to seek care (Table 4).

**Table 4:** Association between symptom presentation and choice of care (No Care/Care)

	**Whole Cohort** (*n* = 174)^a^	**No Care** (*n* = 40)	**Care** (*n* = 134)	***P*-value**^b^
**PCSS Number of Symptoms** *Mean (SD)*	5.7 (3.6)	4.9 (3.3)	5.9 (3.6)	0.054
**PCSS Number of Symptoms** *n (%)*				
1 to 5	106 (60.9)	29 (72.5)	77 (57.5)	0.087
6 or more	68 (39.1)	11 (27.5)	57 (42.5)	-
**Symptoms** *n (%)*				
Headache	142 (81.6)	32 (80.0)	110 (82.1)	0.765
Feeling “foggy”	86 (49.4)	16 (40.0)	70 (52.2)	0.174
Dizziness	76 (43.7)	14 (35.0)	62 (46.3)	0.207
Feeling “slow”	69 (39.7)	10 (25.0)	59 (44.0)	**0.031**
Drowsiness	63 (36.2)	7 (17.5)	56 (41.8)	**0.005**
Nausea	63 (36.2)	9 (22.5)	54 (40.30)	**0.040**

^a^Only those respondents whose symptoms had resolved were asked about their acute symptoms, and thus 174 results are included in this table. Respondents could select more than one symptom, and only the column percentages of each specific symptom (“yes”) are shown (“no” are not shown) and therefore the column percentages do not add to 100%

^b^Difference in PCSS Number of Symptoms (mean) between care groups was assessed using Mann–Whitney U Test. Association between symptoms and care groups were assessed using Chi-squared tests

### Relationship between choice of healthcare and recovery

In this survey, the majority of respondents (86.6%; 174/201) reported that their symptoms had resolved, while 13.4% (27/201) were still experiencing symptoms (Table 5). Distributions of choice of healthcare by symptom resolution, time to symptom resolution and quality of life are provided in Supplementary Table 2. While there was no statistically significant difference in care seeking in those who had symptomatic recovery at the time of assessment, the odds of symptom resolution were four times greater for those who did not seek care compared to those who did (95% CI 0.50–31.98, *p* = 0.19), when adjusted for the type of symptoms experienced (headache, dizziness, nausea, drowsiness, feeling slow, feeling foggy). Of those respondents whose symptoms had resolved, 71.8% (125/174) had taken less than one month to recover. Exploring this further, we identified a significant association between a respondent not seeking care following mTBI and their symptoms resolving in less than one month (Table 5). After adjusting for the number and type of symptoms experienced, the adjusted odds of a respondent experiencing symptom resolution in less than one month were 4.9 times greater for those who did not seek care as opposed to those who did seek care (95% CI 1.51–15.89, *p* = 0.008). The majority (88.4%) of those who did not seek any care selected one or more of the following reasons for not seeking care: “I thought I would be OK” (63%), “My symptoms were not very bad” (37%), or “I decided to watch and wait” (40%).

**Table 5:** Associations between choice of healthcare and (i) symptom resolution, (ii) time to symptom resolution and (iii) quality of life

	**(i) Symptom Resolution**				**(ii) Time to Symptom Resolution**				**(iii) QOLIBRI-OS (Quality of Life)**			
**Type of Care,** *n (%)*	Yes (*n* = 174)	No (*n* = 27)	Crude OR, (95% CI), *p*-value	Adjusted OR^a^, (95% CI), *p*-value	Less than 1 month (*n* = 125)	1 month or longer (*n* = 49)	Crude OR, (95% CI), *p*-value	Adjusted OR^b^, (95% CI), *p*-value	QOLIBRI-OS < 75% (*n* = 120)	QOLIBRI-OS 75% or more (*n* = 81)	Crude OR, (95% CI), *p*-value	Adjusted OR^c^, (95% CI), *p*-value
**No Care**	40(23.0)	3(11.0)	2.39 (0.68–8.34) 0.17	4.0 (0.50–31.98) 0.19	36(28.8)	4(8.2)	4.55 (1.52–13.58) **0.007**^b^	4.90 (1.51–15.89) **0.008**^b^	29(24.2)	14(17.3)	1.52 (0.75–3.11) 0.245	1.32 (0.61–2.86) 0.475
**Care**	134(77.0)	24(88.9)	(Reference group)		89(71.2)	45(91.8)	(Reference group)		91(75.8)	67(82.7)	(Reference group)	
Total Cohort	174 (86.6)	27 (13.4)	-	-	125 (71.8)	49 (28.2)	-	-	120 (59.7)	81 (40.3)	-	-

^a^Adjustment of potential confounder included type of initial symptoms

^b^Adjustment of potential confounder included number of initial symptoms (PCSS total score) and type of symptoms (headache, nausea, dizziness, drowsiness, feeling slow, feeling foggy). The relatively large 95%CI from small number (8% of those who did not seek care had symptom resolution at 1 month or longer) warrants careful interpretation

^c^Adjustment of potential confounder included number of initial symptoms (PCSS total score), type of initial symptoms (headache, nausea, dizziness, drowsiness, feeling slow, feeling foggy) and years of education

Analysis of the QOLIBRI-OS Total score as an indication of quality of life following mTBI revealed minor differences in the mean QOLIBRI-OS score between the types of healthcare accessed. Those who had not sought care had the lowest mean QOLIBRI-OS score (57.2 ± 24.0), whilst the highest QOLIBRI-OS score was 67.4 ± 14.0 for those who had received sports-based care (Table 5). After adjusting for the number of symptoms, type of symptoms, and years of education, the adjusted odds of a respondent having a lower quality of life score were 1.3 times greater for those who did not seek care as opposed to those who did seek care (95% CI 0.61–2.86, *p* = 0.475), albeit statistically insignificant (Table 5).

## Discussion

In this study we investigated the types of healthcare people seek in the early stages following a mTBI in Australia, including seeking no healthcare at all. Due to the lack of centralised health information and surveillance infrastructure in the Australian healthcare system at present, the number of people who experience mTBI and present for care outside of the hospital system is unknown and thus obtaining data from other sources is essential [2, 10, 29]. Our study found that approximately half of the respondents attended a hospital for care, meaning that more than 50% of mTBI reported in this survey would have been missed using the standard method of hospital record surveillance. This has clear implications for estimation of the incidence of mTBI in Australia. Notably, we found that 21.4% of respondents had not accessed any healthcare following mTBI, a rate comparable to previous research conducted in Canada (21.9%) [15], New Zealand (28%) [30] and the USA (30%) [22]. The proportion of respondents who reported attending a primary care practitioner (41%) was slightly higher than that reported in these prior studies, and may be related to variations in healthcare system structure in countries other than Australia [31]. Only 6% of respondents reported accessing sports-based care, and they were often selected in combination with other services, suggesting that these health care providers may provide initial aid but then refer on to either hospital or primary care practitioners.

### Factors influencing choice of care

Compared to data provided by the Australian Bureau of Statistics (ABS), our survey sample appeared to be relatively representative of the Australian population in terms of age (median age of the Australian population is 38 years, compared with 37.7 years in our sample), sex (proportion of females in Australia is 51%, 53% in our sample) and state of residence [32]. However, the respondents in our sample did appear to have a higher level of education overall, with 54% reporting having a Bachelor degree and above compared to 22% reported by the ABS [32]. This may reflect a sampling bias towards respondents of online surveys having a higher level of formal education and being more active online, and may have implications for the interpretation of results relating to education levels in this study as outlined in the limitations section below.

Several demographic, pre- and peri-injury factors, and symptom presentations were found to be associated with the choice of healthcare. Our findings suggest that respondents with a lower level of formal education were less likely to seek care. Previous research has indicated that lower socioeconomic status, including education and income, can adversely influence healthcare access due to barriers such as disparities in health literacy and understanding of the healthcare system, as well as cost and transportation [33, 34]. Those who had experienced a previous mTBI were also less likely to seek care, and it is possible that if an individual knows what to expect following mTBI they are content to self-monitor and manage their condition, as described by Schmidt et al., [35]. Interestingly, those who had experienced previous mental health issues were less likely to seek care, which may suggest that emotional vulnerability may lead to a lower inclination to attend healthcare services. This is consistent with the findings of a Canadian survey of health care-seeking behaviours, who found that in general, patients were less likely to seek care for mental health issues than physical health concerns [36].

The proportion of respondents who reported a loss of consciousness (LOC) in this study (56%) was higher than expected compared with previous literature which suggests that LOC occurs in approximately 8–19% of mTBI [2]. This survey sample was based upon self-reported concussion, and more people may believe they have had a concussion if they have lost consciousness, and conversely, may not identify as having had a concussion if they haven’t lost consciousness [23]. Those who had experienced LOC, as well as memory loss either before or following their injury were more likely to seek healthcare, which suggests that these peri-injury signs cause people enough concern to warrant seeking medical attention. However, as it is possible that this survey sample is biased towards those who had sustained a more significant mTBI, there may be implications with regards to the generalizability of results, as discussed further in the limitations section below.

The most frequently endorsed symptoms (headache, feeling foggy, dizziness, feeling slow, drowsiness and nausea), and least frequently endorsed symptoms (visual problems, vomiting, nervousness, excessive sleep and sadness) in this study are similar to those described previously [24]. If respondents experienced symptoms including drowsiness, nausea, and feeling “slow” they were significantly more likely to seek care. These findings are consistent with previous literature demonstrating that nausea and LOC [16], and LOC and amnesia [23], were the symptoms and signs that prompted most people to seek medical care or advice. Although not statistically significant, those respondents with a low symptom burden (five symptoms or fewer) were less likely to seek care whilst those experiencing more than six symptoms were more likely to attend a hospital ED, suggesting that the self-reported severity of symptoms is a factor in whether professional care is used or not.

A small number of respondents in this survey reported mTBI as a result of intimate partner violence, and these victims likely represent a population group who are considerably under-represented both in terms of mTBI epidemiological research and access to much-needed healthcare resources [20, 21]. Given that these respondents completed this survey voluntarily, it may be that an online survey format may be a safe and feasible method of conducting further research with this vulnerable cohort.

### Relationship between choice of healthcare and recovery

The type of care accessed was associated with recovery time, with a lack of care-seeking associated with recovery in less than one month. However, the relatively large 95%CI from a small number of respondents (8% of those who did not seek care had symptom resolution at 1 month or longer) warrants careful interpretation. Although this finding was unexpected, it was also noted that the symptom burden was lower for those who had not sought care, thus it more likely reflects the initial symptom presentation of the mTBI, rather than being related to the care received. In other words, if an individual’s symptoms were mild they may be disinclined to seek care [23], and experience more rapid symptomatic recovery relative to those with a higher symptom burden.

### Limitations

The primary limitation of this study is that ‘concussion’ was self-reported, without verification from medical records. The use of self-report in this case allowed us to capture the occurrence of mTBI in people who do not seek any care, and provided greater insight into the care seeking behaviours following mTBI in an Australian population. Other authors have suggested that self-report is the most feasible method of data collection for population health surveys, and in particular to acquire information about those who do not access healthcare services following mTBI [15, 23]. However, it is possible that our sample was biased towards those who had experienced more severe mTBI which may have implications for the generalizability of the results. For example, as our sample had a high proportion of respondents who experienced LOC, it may be that the respondents were more likely to seek care, particularly in a hospital setting. Conversely, a sample with a lower proportion of people who had experienced LOC may have an even higher proportion of people who do not seek care.

Selection bias is also likely when conducting online surveys as some demographic groups are more likely to be active online [37]. As outlined previously, the respondents in our study appeared to have a higher level of education overall compared to the Australian population. Our study found that those with a lower level of education were less likely to seek care, and thus it is possible that the actual proportion of people who do not seek care is even higher than the 21% we identified in this sample. Surveys responses were anonymous which means that answers could not be confirmed or clarified, however we felt that respondents would answer the survey more honestly and openly in this format which potentially reduces bias due to ‘social desirability’ [16, 38]. Respondents for this survey were reimbursed by Qualtrics or their panel providers with non-monetary incentives for participating, and this is also acknowledged as a source of potential selection bias [37].

A further limitation of this study is that retrospective data collection may yield less accurate recall of events, particularly with regards to symptom presentation and duration [16]. We selected an 18-month timeframe as we anticipated that this allowed enough time for symptoms to have resolved, but was not so long that the memory of events would be inaccurate. We acknowledge that retrospective data collection has limitations and alternative methods such as shorter timeframes or other case-ascertainment methods could be incorporated in future research. An oversight in our survey design was that we did not specify a timeframe for assessing ‘acute’ symptoms, and we recommend that future studies endeavour to be more explicit in defining a time post-injury. However, we are mindful that, as this was a retrospective study, people may have had difficulty recalling the timeframe of the symptoms they experienced. This is an inherent limitation of retrospective survey-based study designs.

The design of the survey was such that when reporting symptoms respondents were asked “Have you fully recovered from this concussion?” If they selected “Yes”, they were directed to a question asking “What were the main symptoms you experienced following your concussion?”, whilst if they selected “No” they were asked “What symptoms are you still experiencing?” but were not asked about their initial symptoms. Consequently, we did not have data on early symptom presentation for those respondents who had not yet recovered (*n* = 27). In retrospect, we acknowledge that this was an oversight in survey design and would recommend altering this for future studies.

### Significance of the study and future directions

This study demonstrated the feasibility of collecting mTBI information in an online retrospective format to examine patterns of initial healthcare utilisation. The methodology utilised in this survey has the potential to inform the implementation of a large-scale population-based mTBI survey such as an Australian national health survey across different settings. A larger study involving younger (< 18 years old) and older populations (> 65 years old) would provide a more definitive estimation of healthcare utilisation following mTBI in Australia as well as allow collection of further epidemiological information such as trends in age, sex, geographical location and mechanism of injury. In future research, longer term prospective follow-up would provide additional insight into the impact of mTBI on overall health, productivity, employment, and quality of life. Information such as this would assist healthcare providers in planning for the provision of appropriate healthcare resources, as well as the need for more specialised services such as ‘concussion clinics’. Given that a large proportion of respondents in this study attended hospital ED’s and primary care physicians (GP’s) it is also important to ensure that practitioners in these healthcare environments have the relevant skills, resources and confidence in assessing and managing mTBI [39, 40].

Improving community education on the signs and symptoms of mTBI, and the advantages of seeking professional care following injury is also an important step forward to ensure the best possible outcomes for those who have sustained mTBI. In recent years public interest in sports-related concussion has increased exponentially with a concurrent benefit of greater awareness of concussion occurrence [9]. It is imperative that this recognition is translated into better management, ideally through education of key stakeholders including sports players, parents, coaches, sports trainers, medical professionals and official sporting bodies [41]. Community-based seminars and information sessions may be used to build awareness and disseminate current prevention and management guidelines [41]. Technology may also be used to great advantage in the mTBI context, to implement online education courses, social media campaigns and mobile phone-based apps which may assist with concussion recognition and direct people towards early medical assistance.

Capturing information on the types of healthcare that people seek following mTBI in Australia remains challenging, particularly with regards to those cases that do not present to hospitals. This study revealed that over 20% of respondents did not seek healthcare at all, whilst more than half of those who accessed healthcare did so via a hospital emergency department. These findings are important to take into account when estimating mTBI incidence and allocating health resources. Further research efforts should be directed towards accurately quantifying the number of patients who access hospital services nation-wide following mTBI, as well as improving surveillance infrastructure to determine the number of patients who present for primary healthcare outside the hospital environment. By improving our knowledge of the types of care people access following mTBI, including no care, we will gain a better understanding of the extent of mTBI in Australia. 

## Supplementary Information

**Additional file 1:** **Supplementary Table 1. **Variables used in this study with corresponding survey questions, possible responses and re-categorisation.

**Additional file 2:** **Supplementary Table 2. **Distributions of choice of healthcare by (i) symptom resolution, (ii) time to symptom resolution and (iii) quality of life.

## Data Availability

The datasets generated and/or analysed during the current study are not publicly available due to ongoing data analysis as part of the *“Recovery Experiences Following Concussion Survey”*, but are available from the corresponding author on reasonable request.
