# Supplementary material for: Healthcare choices following mild traumatic brain injury in Australia
Source: BMC Health Serv Res. 2022 Jul 4;22:858. doi: 10.1186/s12913-022-08244-3 (PMC9254542; doi:10.1186/s12913-022-08244-3)
Supplement: Supplementary file 1 — Additional file 1: Supplementary Table 1. Variables used in this study with corresponding survey questions, possible responses and re-categorisation. [file 12913_2022_8244_MOESM1_ESM.docx]

**Supplementary Table 1: Variables used in this study with corresponding survey questions, possible responses and re-categorisation**

| **Variables included in statistical analysis** | | | | |
| --- | --- | --- | --- | --- |
| **Variable** | | **Survey Question** | **Responses** | **Re-categorisations (if applicable)** |
| **Demographic Factors** | | | |  |
| Age | What is your age? | | - Value between 18 and 65 |  |
| Age (categories) | What is your age? | | - 18-25 - 26-35 - 36-45 - 46-55 - 56-65 |  |
| Sex | What is your gender? | | - Male - Female - Other - Rather not say |  |
| State | What is your state of residence? | | - Australian Capital Territory - New South Wales - Northern Territory - Queensland - South Australia - Tasmania - Victoria - Western Australia |  |
| Education Status | What is your highest level of completed education? | | - Secondary to Year 10 - Secondary to Year 12 - Vocational Training - Diploma - Bachelor’s degree - Post-Graduate Studies | Years of education calculated by assigning the following years:   - Secondary to year 10 = 10 years - Secondary to Year 12 = 12 years - Vocational training = 13 years - Diploma = 14 years - Bachelor’s degree = 15 years - Post-graduate studies = 18 years |
| Employment Status | What is your employment status? | | - Full-time - Self-employed - Part-time - Home Duties - Currently not working |  |
| Household Income | What is your household income level? | | - Nil - $1-$49,999 - $50,000-$99,999 - $100,000-$149,999 - $150,000-$199,999 - $200,000+ | - < $100,000 - ≥ $100,000 |
| **Most recent concussion** | | | |  |
| Symptom Resolution | Have you fully recovered from this concussion? | | - Yes - No |  |
| Time to symptom resolution | Can you estimate how long it took for your symptoms to resolve? | | - <2 weeks - 2-4 weeks - 1 to <3 months - 3 to <6 months - 6 to <12 months - 12 to <18 months | - < 1 month - > 1 month |
| PCSS Acute Symptoms | What were the main symptoms you experienced following your concussion? (Please select all that apply) | | - Headache - Nausea - Vomiting - Balance problems - Dizziness - Fatigue - Trouble falling to sleep - Loss of sleep - Drowsiness - Light sensitivity - Noise sensitivity - Irritability - Sadness - Nervousness - More emotional - Numbness or tingling - Feeling ‘slow’ - Feeling ‘foggy’ - Difficulty concentrating - Difficulty remembering - Visual problems | Total number of symptoms:   - <6 - 6 or greater |
| Injury Description | Can you please provide a short description of what happened to cause your most recent concussion? | | - Open field response |  |
| Injury Cause | What activity was your concussion related to? | | - Sport - Non-sport |  |
| Non-sport Type | If your concussion was not related to sport, what was it related to? | | - Fall - Transport accident - Assault - Other (open field) |  |
| Loss of Consciousness | Did you lose consciousness during the incident that caused your most recent concussion? (Were you “knocked out” or did you “black out”?) | | - Yes - No - Unsure |  |
| Memory loss prior | Did you experience any memory loss for events immediately before this concussion occurred? | | - Yes - No - Unsure |  |
| Memory loss after | Did you experience any memory loss for events immediately after this concussion occurred? | | - Yes - No - Unsure |  |
| **Past medical history** | | | | |
| Previous mTBI | Have you had any previous concussions (apart from the most recent injury described above)? | | - Yes - No |  |
| Previous medical issues | Have you experienced the following medical issues either before or after your concussion?   - Epilepsy or seizure disorders (“fits”) - Migraine or other headache disorder - Sleep disorders (insomnia, sleep apnoea) - Learning disorders (dyslexia, ADHD) | | - Yes - No |  |
| Mental health issues | Have you ever been diagnosed with a mental health disorder (such as anxiety, depression, panic attacks, post-traumatic stress disorder, other psychiatric disorders)? | | - Yes - No |  |
| **Choice of Acute Care** | | | | |
|  | When you first experienced your most recent concussion, where did you seek medical care? (select all that apply) | | - None, I did not seek medical care at all - Public Hospital Emergency Department - Private Hospital Emergency Departments - General Practitioner (GP) - Urgent Care or After Hours Medical Centre - Sports Doctor/Sports Physician - Sports Club Personnel (Doctor, physiotherapist, sports trainer) - Other (please give details) | - No care - Hospital ED - Primary care - Sports-based care |
|  | If you did not seek any medical care, what were your reasons for this? (select all that apply) | | - My symptoms were not very bad - I thought I would be OK - I decided to “watch and wait” - I didn’t know where I should go - I couldn’t get an appointment - Someone else made the decision for me as I was not thinking clearly - I didn’t want others to know that I had experienced a concussion - Other (please provide details) |  |
| **Quality of Life** | | | | |
| QOLIBRI-OS | We would like to know how satisfied you are with different aspects of your life since your concussion injury. For each question please choose the answer which is closest to how you feel now (including the past week):  Overall, how satisfied are you with your physical condition?  Overall how satisfied with how your brain is working, in terms of your concentration, memory and thinking?  Overall how satisfied are you with your feelings and emotions?  Overall, how satisfied are you with your personal and social life?  Overall, how satisfied are you with your current situation and future prospects? | | Likert Scale 0-4:   - 0 Not at all satisfied - 1 Slightly satisfied - 2 Moderately satisfied - 3 Quite satisfied - 4 Very satisfied | QOLIBRI-OS score:   - Lower QoL score (Less than 75%) - Higher QoL score (75% or greater) |
