# Supplementary material for: Healthcare choices following mild traumatic brain injury in Australia
Source: BMC Health Serv Res. 2022 Jul 4;22:858. doi: 10.1186/s12913-022-08244-3 (PMC9254542; doi:10.1186/s12913-022-08244-3)
Supplement: Supplementary file 2 — Additional file 2: Supplementary Table 2. Distributions of choice of healthcare by (i) symptom resolution, (ii) time to symptom resolution and (iii) quality of life. [file 12913_2022_8244_MOESM2_ESM.docx]

**Supplementary Table 2: Distributions of choice of healthcare by (i) symptom resolution, (ii) time to symptom resolution and (iii) quality of life**

|  | 1. **Symptom Resolution** | | 1. **Time to Symptom Resolution** | | 1. **QOLIBRI-OS (Quality of Life)** | | |
| --- | --- | --- | --- | --- | --- | --- | --- |
| **Type of Care,**  *n (%)* | Yes  (n=174) | No  (n=27) | Less than 1 month  (n=125) | 1 month or longer  (n=49) | QOLIBRI-OS Total Score  *mean (SD)*  (n=201) | QOLIBRI-OS <75%  (n=120) | QOLIBRI-OS 75% or more  (n=81) |
| **No Care** | 40(23.0) | 3(11.0) | 36(28.8) | 4(8.2) | 57.2 (24.0) | 29(24.2) | 14(17.3) |
| **Care** | 134(77.0) | 24(88.9) | 89(71.2) | 45(91.8) | 63.2 (25.5) | 91(75.8) | 67(82.7) |
| - Hospital ED | 81 (46.6) | 15 (55.6) | 47 (37.6) | 34 (69.4) | 63.0 (25.9) | 54 (45.0) | 42 (51.9) |
| - Primary Care | 62 (35.6) | 13 (48.1) | 47 (37.6) | 15 (30.6) | 62.2 (25.8) | 45 (37.5) | 30 (37.0) |
| - Sports-based Care | 9 (5.2) | 3 (11.1) | 4 (3.2) | 5 (10.2) | 67.4 (14.0) | 7 (5.8) | 5 (6.2) |
| Total Cohort | 174 (86.6) | 27 (13.4) | 125 (71.8) | 49 (28.2) | 61.9 (25.3) | 120 (59.7) | 81 (40.3) |
